# A Treatise on the Nature and Cure of Those Diseases Which Precede Change of Structure

**Published:** 1830-10-01

**Authors:** 


### A Treatise on the Nature and Cure of those Diseases, either Acute or Chronic, which precede Change of Structure; with a View to the Preservation of Health, and particularly the Prevention of Organic Diseases. By *A. P. W. Philip*, M.D. &c. Octavo, pp. 432. Longmans, 1830.

We entirely agree with Dr. Philip in the following sentiment, with which he opens his work :—" I believe a physician who has been long engaged in practice, cannot better promote the objects of his profession, than by simply relating, with accuracy, the facts he has himself observed, and the reflections they have suggested." This we believe, provided the execution be conformable. But if, in laying open this experience and these reflections to the world, we hope or expect to make the non-professional public anatomists, physiologists, and physicians, we shall lamentably expose the vanity of our enterprize. In such an undertaking Dr. Philip embarked, when, a few years ago, he attempted to make "the laws of the vital functions"—laws which are very imperfectly known to physiologists themselves —a part of general science, and consequently the study of general readers. These are his own words, in the preface before us. " In the introduction to the third edition of my Treatise on the Vital Functions, which may rather be considered as belonging to general science than exclusively to medicine, I gave such a view of the functions of the animal body as would enable the *general reader to understand all that is said in it, and, consequently, in the present Treatise.* I have endeavoured, however, to render the practical, that is the second part of this Treatise, intelligible to those who do not possess even that knowledge of our frame." Thus in even an Introduction, Dr. P. conceives that he has enabled the *general reader* to understand *all* the " laws of the vital functions"—while the second part of the present work—namely, the *treatment* of diseases is rendered, or supposed to be rendered, intelligible by the same class of readers, even without the knowledge of the vital functions.

Now, the total failure of the first enterprise, as his publisher's shelves can attest, might have moderated Dr. Philip's zeal in the illumination of the public, or even checked the overflowings of his philanthropy in rendering them independent of doctors as well as of diseases—for we will venture to affirm that not 20 copies of the "vital functions" ever travelled beyond the boundaries of the profession—except as donatives—and that not twenty individuals ever read—nor one understood, a fiftieth part of these vital laws. No verily! The science of life is studied in a very different manner by the public at large. They are much more inclined to pick up its precepts in the "Heart of Mid Lothian," or "Tom and Jerry," than in the dissecting-room, or in the fair pages, of Dr. Philip's "third edition." We should like to hear Dr. P. catechise his brethren of the Royal Society respecting the "laws of life:"—*some* of them, at least, would answer him in the laconic words of an ancient poet—

" *Vitam* faciunt Balnea, Vina, Venus."

The Royal Society indeed, and the Quarterly Review, appear to have turned the brains of two very clever physicians—Drs. Philip and Uwins—for ever since the said SOCIETY and the QUARTERLY did these physicians the "*honour*" of publishing their lucubrations, the ears of the profession have been stunned with the eternal reiterations of the said "honours." Our author informs us that though he was a compiler from the works of others, in early life—(and we think a very respectable one, as evinced by his Treatise on Febrile Diseases,) yet he has "made use of none in the composition of this Treatise"—that is, none but his own—these being now so numerous as to form a sufficient library of reference for himself at least. And truly Dr. Philip is by no means chary in his references to former publications. The procedure is extremely politic, and is one of the most ingenious and clever modes of evading the advertisement duty, with which we are acquainted. It perpetually jogs the flagging memory of the public towards our past labours—for no writer knows better the truth and force of the following precept than Dr. Philip.

Men must be taught as if you taught them not,
And things *long known* proposed as *things forgot.*

"In my late publications, my objects have been to state my own observations, and the inferences to which they have led me: and, as *many parts* of the present Treatise relate to the subjects of these publications, the various papers which the Royal Society have done me the honour to publish, a Treatise on the Vital Functions, and that on Indigestion, it was necessary either to repeat what I had already published, and swell this volume to too great a size, or frequently refer to them. This will explain my *frequent reference* to them, and, I hope, be my apology with the reader." *Pref.* x.

Whether this frequency of reference indicates any secret misgiving in the mind of the worthy author, that the MEMORY of the public is a treacherous jade, or rather JILT, ever ready to coquet with a new, and forget an old face, we shall not venture to determine; but if these frequent references induce the readers to drink at the fountain head, and *consult* the originals, Mr. Underwood may set up his carriage at once.

Dr. Philip forewarns us that "he shall indulge in no speculative doctrines, but wholly confine himself to facts which came under his own view, *and the necessary inferences from them.* Under this last title, the author knows well that he may speculate as widely as he pleases. He "avoids the narration of cases"—though these are the most substantial kind of facts—and as "the *purposes* (of this Treatise) would be very *imperfectly answered*, were it not made intelligible to the GENERAL READER, he shall, as far as he can, avoid the use of *technical language*." And has the lecturer, the experimental physiologist, the long-established practitioner, the wealthy and independent physician, condescended, at his time of life, to throw aside the language of medical science—to turn his back upon the profession—and address the mob?—And for what *purpose?* For the avowed purpose of enabling general readers to comprehend all that he has written on the vital functions, on indigestion, and in the present Treatise! Yes! To make the non-professional public intimately acquainted with the symptoms and the treatment of those obscure, proteiform, and almost incognizable deviations from sound health, or approaches to disorder, which often elude the eye of the most observant physician, or baffle his skill when detected, is the professed object of the volume before us! We can scarcely reconcile such an extravagant, unfeasible attempt, with that prudence which generally accompanies grey hairs—and Heaven forbid that we should suspect Dr. Philip of having any other object than the one which is proclaimed. But, while we absolve the author from all selfish *motives*, we must and do impugn his judgment, in thus appealing from the profession to the public at large—and in thus attempting to force the study of diseases and the methods of treating them down the throats of non-professional readers.

A physician may now, as did Hippocrates, of old, write popularly on various points of hygiene, such as the salubrity or insalubrity of the air we breathe, the food we eat, the water we drink, the raiment we wear, the avocations we pursue, &c. These are subjects in which all are concerned, and which all may study; but if a physician writes a treatise on the nature, causes, and treatment of diseases of the brain, the heart, the lungs, the liver, and the stomach—and tells the general reader that he has rendered *all* that is in his book perfectly comprehensible by him, without the labours of the dissecting-room, and the bad air of hospitals,—then we say that the physician who believes that he can do what he thus promises, must be insane—if he do not believe it, he is, to say the least, disingenuous.* But we do not think that Dr. Philip is either insane or knavish—he is only very CUNNING. He has lived long enough in this world to know that flattery is the best unction for the soul—and that a little innocent flattery applied to the understanding of the public at large, will greatly prepossess them in favour of his lucubrations—without doing the least harm, but probably good to the profession at large, since it is not very likely that general readers will make better general practitioners than those who have been all their lives at the bed-side of sickness—indeed it is reasonably to be expected that, should the Doctor's book become epidemic in this country, the medical practitioner will seldom have an opportunity of prescribing for those "diseases which precede change of structure," since the organic affections will be all ready prepared for him by the patient, upon the soundest canons in the Philippian code of health and longevity.

We should not indeed be much surprized if Dr. Philip was all this time laughing in his sleeve at the HOPES which he has excited in the general public that they will get rid of the Doctors—and the FEARS which he may call forth in the professional republic, of losing their practice—well aware that his work will disappoint both parties—the latter somewhat agreeably by adding considerably to their revenue.

A medical writer, who addresses himself unequivocally to the general reader, and, adapts his language and consequently his materials to the non-professional scale, cannot expect a very extensive perusal by his own profession, who look to a higher species of information than can be conveyed to the patients whom they attend. It cannot, indeed, have entered into the designs or wishes of Dr. Philip that his book should circulate within the pale

* We ask Dr. Philip this simple question:—if the general reader can understand *all* that is contained in his treatise, respecting diseases and their cure, why should that general reader apply to him or to any other medical man, for knowledge or advice which he already possesses?

of medical society—for he, at least, must be conscious that almost the whole of the present treatise is composed of fragments from his former works, sometimes cemented together very roughly—sometimes melted down, and the same materials moulded merely into new forms, on the approved recipe of the Roman poet—

> In nova, fert animus, mutatas dicere formas,
> Corpora.

To say the truth, Dr. Philip is not singular in this method of multiplying literary bantlings without any new births, but merely by fresh christenings; yet such procedure is very disadvantageous to medical literature, already overloaded with masses of useless, indeed cumbrous materials. On this account, we are well aware of the extreme difficulty which we should encounter in making even a tolerable article for our review out of 430 pages of goodly octavo. Still we must notice the book—since any work emanating from so justly esteemed a writer as Dr. Philip, even when addressed to the populace, is entitled to examination.

The first part of the work is on the nature and symptoms of those derangements which precede change of structure—a strange and quaint title for a book, since it must apply to, or include almost the whole range of nosology. This, however, is of little consequence.

Dr. P. observes, that many people mix in society, under chronic affections, which steal on by degrees, and thus produce a gradual accommodation of the feelings and functions to a state which, had it suddenly occurred, would have unfitted the individual for social intercourse with the world. These incipient deviations from health are sometimes the consequence of acute attacks of disease—sometimes arising from imperceptible beginnings. They are classed by our author under the three following heads :—

"General debility accompanied by a state of inanition, the consequence of a general failure of power in the various processes by which our food is converted and properly distributed ; general debility accompanied by a state of plethora, from a failure in the power of those organs which throw off from the system what is no longer necessary, and soon becomes injurious to it ; and, lastly, a greater or less failure of function in some particular organ essential to life." 6.

The first of these states is considered by Dr. P. as extremely rare, as a permanent affection. It occurs occasionally after the severe shock of an acute disease, when the system appears not to receive due nourishment. This state lasts not long. The patient either rallies, or some local disease is set up in the weakest part.

"General debility accompanied by plethora, on the contrary, is one of the most common as well as insidious forms of permanent chronic disease. I regard it as a state of general disease, because it affects equally every part of the system ; although, in considering its causes, it will appear that in most cases, it is in the first instance, a disease of the excreting organs." 8.

Those who are acquainted with the writings of the late Dr. Parry, will remember that the above is one of his fundamental principles of pathology, and is not, therefore, so original a proposition as Dr. Philip may suppose. We have indeed generally remarked, that those who consult least the works of others, shew least originality in their own writings. The reason, we think, is obvious. Acquaintance with what has been put on record enables us to discriminate what is original from what is second hand.

Dr. Philip justly remarks that although the animal body is fruitful in resources, it cannot provide against all contingencies. One man may eat and drink double as much as his neighbour, and may still be in as good health, if his excreting organs are in a state of activity, and carry off a proportionate superfluity of nutriment. But the pleasures of the table, in civilized life, not only provoke to too much ingestion, but, by imparing the tone of the excretory organs, check the salutary office of the waste pipe. There is some questionable pathology in the following quotation.

"Contrary to what we should at first view suppose, it is in those who have naturally the smallest appetites that states of plethora are most apt to arise. Except as far as we are influenced by a wish to gratify the palate, our desire for food is proportioned to the demand for it. In those constitutions where the excreting organs—and particularly the skin, which is the most extensive—are in a state of greatest activity, the greatest supply is required, and any morbid accumulation least apt to arise. In those whose excreting organs are languid, a less supply is necessary, and an accumulation more apt to take place. Those who naturally *eat little*, for the same reason, are generally also *fatter* than those who eat a great deal." 10.

There can be no doubt that those who use active exercise, and whose skin and secreting organs are in full force, require and bear much more food than those who are weakly; but we ask Dr. Philip, is it generally among those weakly persons, with puny appetites, that we see apoplexy, aneurisms, hypertrophy of the heart, and other unequivocal states of great plethora? The last part of the quotation would seem to infer that obesity is the consequence of little eating—whereas we should be more inclined to reverse the proposition. The fact is, that idleness and good living will cause obesity, or some disease in the majority of people—and, on the other hand, paucity of food will generally lead to paucity of fat.

Passing over a number of trite and obvious truths, which it must have been wearisome to write, and would be unpardonable to repeat here, we can only now and then catch a passage capable of exciting the slightest attention.

"The chief indications of a plethoric state of the system are a languor and sluggishness both of mind and body, and often a distressing sense of debility even in slight exertions. Those parts of the body where the vessels are most superficial, the cheeks, eyes, &c., often become redder than usual; and flushings, often followed by some degree of perspiration, are not unusual.

But a state of plethora may exist without these latter indications; for it seems sometimes to happen that, without any determination of blood to a particular organ, the more internal vessels become loaded, while those of the surface appear no more charged with blood than usual: and the opposite of this state sometimes exists; the external vessels being more charged than those of internal parts, and a red and turgid countenance, which suggests the idea of threatened apoplexy, will continue for many years; and that, where the causes of plethora continue to be applied without symptoms of internal disease, *and seems even to be a means of prevention*—the external vessels affording a receptacle for the superabundant blood, and thus tending to prevent morbid distention of those more internal." 16.

We suspect there is more of fancy than of fact in the idea that a red face, turgid countenance, injected vessels, &c. are not merely indications of an easy circulation internally, but preventives of plethora in the vessels of the viscera. True, we see men go about for years, with the turgid faces alluded to; but the question is, are such people less subject to apoplexy than the pallid and meagre-visaged? We apprehend that very few will draw the *inference* which Dr. Philip has drawn. We were going to call it a theory —but Dr. P. has declared that he indulges in *no speculations*. Nothing but facts!

Dr. Philip, indeed, seems to delight in dwelling on the exceptions to general rules, rather than on the general rules themselves. Thus, at page 25, he says :—

> "In healthy habits, although the immediate effects of blood-letting is to lessen the quantity of blood, its tendency, by the general check it gives to the action of the various excreting organs, is to increase it. Thus butchers bleed animals to fatten them."

One or two bleedings, in healthy habits, might have such effects as are above stated; but let the healthiest man lose an ounce or two of blood daily from the hemorrhoidal vessels, or other outlet, and we shall see whether he will fatten or get thin and pallid—perhaps dropsical by the bleeding. Although Dr. P. has addressed his work to general readers, we are confident that not one in one hundred of them will understand a word of what he has said about plethora, and congestion, and capillary debility, &c. in his first chapter. The same remark is still more applicable to the second chapter, on "The Powers of the Nervous and Vascular Systems, and the Relation they bear to each other." What he has here laid down is known already to physiologists—and never can be beaten into the heads of general readers —who, indeed, would be great fools for studying any such matters. The "Vital Functions," however, and the "Philosophical Transactions" have enabled Dr. Philip to fill up very easily 54 pages of letter-press, constituting the second chapter of the work before us.

The third chapter is on "Some of the More Acute Diseases of the Brain."

Our author divides the occasional causes of diseases of the brain into those which morbidly excite, and those which morbidly depress its powers. He then takes a very cursory view of the effects of these two sets of causes.

> "When the brain is exposed to a highly exciting cause, from the immediate influence of this organ on the heart and blood-vessels, they also are excited to increased action. If the cause be of a transitory nature, and not excessive, little other sensible effect ensues. As its influence subsides, the functions of the brain and sanguiferous system return to their usual state.
>
> When it is excessive, though of short duration, other consequences sometimes ensue. The blood is conveyed to the head by vessels subject, of course, to the same affections as those of other parts of the body. When the sanguiferous system, therefore, is preternaturally excited, they partake of this increased excitement; and by this cause, combined with the increased action of the heart, a greater than usual quantity of blood is sent to the head: but as the blood is returned from the brain by membranous canals which cannot partake of this excitement, a tendency to accumulation of blood in the head takes place; which is much increased, if the occasional cause has been of such a nature as at the same time to excite the muscles of voluntary motion; whose action, by pressing irregularly on the veins, in consequence of the valvular structure of these vessels, greatly increases the rapidity of the circulation.
>
> An increased accumulation of blood in the brain, within certain limits, for the time increases its energy; and both the valvular structure of the veins in the limbs, and the inexcitable nature of the canals just mentioned, appear to have for their object, that the vigour of the brain should be temporarily increased under strong exercise, and the influence of certain passions, in proportion to the increased demand for it. This temporary increase of excitement in the nervous and sanguiferous systems is succeeded by a proportional depression in the powers of both.

It sometimes happens, during the state of excitement and consequent turgescence of the vessels of the brain, that one of these gives way; and, blood being effused on the surface or in the internal parts of the brain, this organ is so compressed as to become suddenly incapable of its functions, and one of the most fatal forms of apoplexy ensues.

When, without rupture of the vessels, the brain is so compressed by their morbid distention as to become incapable of its functions, an apoplectic state in like manner ensues; but which after the cause of the excitement is removed, if the constitution be otherwise sound, disappears spontaneously. I knew an elderly gentleman, stout, and of a full habit, who laboured under hooping-cough, and every return of cough left him in a state of insensibility on the floor. He, however, passed through the disease without any serious accident, and enjoyed his usual health after it.

When such distention of the vessels arises from more permanent causes, or the vessels of the brain have been previously debilitated, the result is more serious, and constitutes one of the most frequent forms of apoplexy; the recovery from which depends on the vessels, when being relieved from the superfluous quantity of blood, recovering and maintaining their vigour, and consequently their healthy diameter. But it is not unusual, when a plethoric state of the head has been habitual for some time previous to the apoplectic attack, and the vessels consequently have been debilitated, for the patient several times to revive on abstraction of blood; but constantly to relapse into the same state, as the vessels again allow themselves to become morbidly distended, till, the powers of the constitution being exhausted, death closes the scene." 91.

From this extract—one of the most strictly medical in the work—our readers will see a specimen of that dead sea through which we are obliged to wade, in search of what might be reasonably expected from the pen of an eminent English physician in the nineteenth century. From page 90 to page 136, we have not been able to find a single sentence which would be worth the trouble of transplanting to our pages—and how the talented writer was able to weave so long a tissue of narcotism, as the present volume presents, without falling into irrecoverable coma, we are utterly at a loss to conjecture. If general readers can study the 3d, 4th, and 5th chapters of the work before us, without falling into a profound sleep, they may take, with safety, but without any effect, a full ounce of Battley's XX liquor opii sedativus.

The 6th chapter is on the Morbid Affections of the Heart which precede Change of Structure.

The heart, says Dr. Philip, "has but one function, that of impelling the blood." "A necessary consequence of the simplicity of its structure and its function is, that, *its diseases are also simple.*"

"They may be divided into two classes,—those which weaken the power with which it propels the blood, and those which impede the passage of the blood through it. The former, the diseases of the substance of the heart itself; the latter, of its orifices and its valves." 137.

Whether the heart be incapable of propelling the blood from weakness of its structure, or imperfection of its valves, the effect must be a *diminution* of the force of the circulation. And thus the immense class of diseases arising from hypertrophy of the heart, and inordinate impulsion of the blood, is, at once, swept from the chart of pathology—and that by a metropolitan physician of the present time, who never once even alludes to auscultation in his treatise ! ! It would be an insult to our readers to dwell, for a moment, on this astounding proof that Dr. Philip has ceased to keep pace with the progress of science, when he has ceased to consult the labours of his cotemporaries. This is one of the blessed fruits of wrapping ourselves up in self-experience—or rather self-conceit, and shutting the eye and ear against every thing that is going on beyond the circle of our own practice. Is it not astonishing that men will come forward in the character of authors, who tell us they never read—and consequently (we must infer) that they contemn reading—and yet modestly expect that all others shall read *their* productions ! We entreat the reader's attention to the following extract.

"In such a treatise as the present, whose objects are, to detect the *first beginnings of organic disease,* and point out the means of obviating them, it would be of little advantage to dwell on the *simple organic diseases* of the heart; *because they betray themselves by no symptoms till they have made such progress that we have no means of arresting them :* and *the same observation applies to the organic diseases of the aorta, and other large vessels attached to the heart.*

But although we have no warning of the approach of simple *organic disease of the heart,* and therefore can lay down no rules for its prevention, as *organic disease* is sometimes the effect of *other diseases of this organ,* which may *both be detected and relieved,* these diseases are the proper subjects of such a treatise." 137.

In the whole course of our lives we never read such a jumble of absurdity, contradiction, and *bad physic,* as the foregoing extract contains. We were first told that the diseases of the heart were *simple* (certainly a very simple enunciation) on account of the simplicity of its function and structure. In the very next page, we are informed that simple organic diseases of the heart (the only ones that can exist according to the preceding proposition) *betray themselves by no symptoms* till they are irremediable;—and then we are told that *organic disease* is sometimes the effect of *other diseases of this organ, (!)* which can be detected and relieved! Not the least notice is taken of that gradual accretion of the pericardium to the heart, or of that gradual increase of thickness in the parietes of the heart, producing such fearful and injurious disturbance of function in various other organs of the body, and ultimately death. Not a word is said about the detection of these and many other insidious diseases by auscultation. No! A few trite observations on inflammation of the heart, taken chiefly from his own compilation made 30 or 40 years ago, in Edinburgh, fill up the remainder of this sixth chapter, and offer a lamentable specimen of British pathology, diagnosis, and practice in the year 1830!

We now come to the 7th chapter of the work, on disease of the lungs. Dr. Philip observes that it has been thought by some that tubercles may sometimes arrive at a state of magnitude or growth which will defy our remedies, without producing any symptoms by which they can be detected.

" If this ever happen, which I greatly doubt, it must be very rarely; because I have found that, in the *most consumptive habits,* the first symptoms can generally be checked, and *perfect health* re-established." 155.

This indeed is consolation! Perfect health can be brought about in *the most consumptive habits*, by Dr. Philip, when the early symptoms are seen by our author! Dr. P. does not believe that those predisposed to consumption are born with the seeds of tubercles in their lungs. It would be vain to tell him that the actual tubercles themselves have been detected in the lungs of the fœtus in utero, and of the infant newly born.

"We may, therefore, very confidently assume—and at all events they are the safest assumptions—*that disease of the lungs never exists without betraying itself by evident disorder of their function*, and that, at the commencement of the symptoms, the lungs contain neither tubercles nor their seeds. And the opposite assumptions are so gratuitous, that, as far as I know, not even an attempt has been made to adduce a direct proof of either. *Tubercles, I believe, are always the consequence of some occasional cause, and, in the first threatenings of the disease, may generally be prevented*, however strong the disposition may be, by correcting the symptoms which precede them." 156.

Is it not rather surprizing that disordered function of the lungs should be evident when disease exists there; while in the heart, which, according to our author, is far more simple in structure and function, the *organic diseases* "betray themselves by *no symptoms* till they have made such progress that we have no means of arresting them." This is surely one of the most palpable contradictions that can be imagined. The reason no doubt is, that Dr. P. has paid no auscultic attention to the *physical signs* of disordered function or structure of the heart, and consequently knows nothing of them. But by attention to the lungs, he thinks he can tell the early symptoms that are and that are *not* attended with tubercles.

That tubercles are always caused by some local disease of the part where they are found, and never rise from an hereditary taint, we do not believe—and Dr. Philip has not advanced the shadow of a proof to that effect. Much do we doubt, too, the power which we are said to have over the formation of tubercles.

Some vague and rambling observations follow on disease of the lungs produced by drunkenness—and then the old subject, "dyspeptic phthisis," from neither of which can we glean any new information. Alluding to the tabes of drunkards, Dr. P. remarks:—

"*Pulmonary consumption*, and the disease we have just been considering, are the most common *causes* of organic disease of the lungs; but, as in all other organs, long continued derangement of function, from whatever cause, will sooner or later have this effect." 168.

We would ask Dr. Philip what is "*pulmonary consumption?*" Is it not an organic disease of the lungs? No, says he—it is only one of the most common *causes* of organic disease? Such reasoning was never before heard. A disease is made to be its own cause!

### Chap. VIII. On Organic Disease of the Stomach.

Considering how much Dr. Philip has attributed to an inflammatory process going on in the stomach, as the cause or concomitant of indigestion, and how long he has patronized those active little auxiliaries to physic and surgery—leeches—we were rather surprized to learn, from our author's farther experience, that the stomach is nearly insusceptible of organic disease.

"Of all the vital organs, the stomach is least prone to organic disease. It seems almost to form an exception to the general law, that long continued derangement of function terminates in disease of structure: for we see people labouring under a certain degree of indigestion for the greater part of life, and even the more severe forms of it, without apparently any *tendency to organic disease of this organ;* and that even when the irritation of the stomach has been such as, through the intervention of the nervous system, to derange the structure of distant parts; and, on the other hand, when organic diseases of the stomach do take place, it is not particularly in those who have been troubled with symptoms of indigestion." 174.

Never was there devised a more ingenious theory than this. It is absolutely impregnable. The stomach may disorganize the most distant parts of the human frame, but it is proof against alteration of structure itself. The diagnosis therefore never can be corrected by the scalpel—for whether disease be found or not, the Doctor and the doctrine are securely entrenched against all attacks. We do not indeed deny that disordered function of the stomach may derange the functions of the brain and various other parts of the machine; but we have great doubts of the extent of power in disorganizing distant structures, which Dr. P. is inclined to confer on it. The following passage is quite unobjectionable.

"From the extreme sensibility of the stomach, and the various causes of irritation to which it is exposed, there is hardly any part of the body that is not liable to be affected through it; and it gives peculiar power to all its sympathies, that the brain, through the medium of which all sympathetic affections take place, peculiarly sympathises with it. Thus it is, that by indigestion the whole system is kept on the fret, if I may use this expression; and if any part is more liable to disease, of whatever nature, than the rest, it is apt to show itself." 177.

### CHAP. IX. ON MORBID AFFECTIONS WHICH PRECEDE CHANGE OF STRUCTURE IN THE LIVER.

The opening sentence of this chapter convinces us, if there were no other proofs in Dr. P.'s works, that the author is very little acquainted with morbid anatomy. "Of all the vital organs, next to the lungs, it (the liver) is the most frequent subject of organic disease." Now we fearlessly appeal to every practical anatomist, hospital physician, or even to the students at hospitals, whether there ever was uttered a more erroneous sentence than the above. Take, for example, the heart, and we confidently affirm that, for one organic disease of the liver which we meet with in the dead-house of an hospital, we shall find six, if not more, of the central organ of the circulation. One would suppose, indeed, that Dr. Philip drew his conclusions from the inconsiderate and inaccurate phraseology of routine practitioners, or even patients themselves, rather than from the dissecting-room. No term is more common in the mouths of *routinists* than "LIVER COMPLAINT"—yet in not one-twentieth of these "liver complaints" is there any disease of the organ at all! That the function of the liver, and the condition of the bile, are very often deranged, we can have no doubt; but those who are candid will admit that we are very imperfectly acquainted with the nature of these functional derangements—and that we often infer their existence upon very doubtful evidence—or rather without any good evidence at all.

That the sympathy between the stomach and liver should be one of the most common and most powerful, we have every reason to believe—even from their direct association in one mutual process—digestion. Dr. Philip avers, and we think truly, that "derangement of its function (the liver) almost uniformly attends that of the stomach"—but how does the following passage accord with our author's general doctrine of the stomach's power to disorganize those viscera with which it is linked in sympathy?

"In this country at least, indigestion, however severe and long-continued, seldom produces disorganisation of the liver, except in drunkards and those who have suffered from the effects of sultry climates." 180.

The following quotation will add another proof that Dr. Philip does not keep pace with modern advances in pathological science.

"It is not at all uncommon for blows on the head to produce inflammation of the liver; an effect they rarely, *if ever*, produce in any other of the abdominal or thoracic viscera; and, on the other hand, while, in the most severe inflammation of the heart, lungs, stomach, or bowels, the mental functions remain clear to the last, delirium attends that of the liver." 181.

It has been well ascertained, of late years, that blows on the head, or in any other part of the body, together with fractures and surgical operations, more frequently produce purulent depôts (which must be what Dr. P. means by inflammation) in the *lungs* than in the liver. Neither can we coincide with our author, that delirium is such a common attendant on acute hepatitis—and so invariably absent in other inflammations. Surely Dr. Philip could not have witnessed many cases of acute carditis, or he would not have made the hardy assertion, that the sensorial functions remain unaffected till the last. It is curious, if this delirium be such a constant attendant on hepatitis, that Dr. Good never once mentions the phenomenon under the head of Empresma Hepatitis—while he dwells on the delirium which attends certain forms of pleuritis, and which, indeed, gave occasion to the term *paraphrenitis* by the ancients, and even by the moderns. Every practitioner, in fact, knows, that whenever the general febrile action kindled up by a local inflammation, wherever situated, rises to a certain degree of intensity, the sensorial functions are drawn into the circle of disorder;—and it is inconsistent with accurate observation to lay it down as a dogma, that inflammation of the liver has the exclusive privilege of disturbing the functions of the brain in the form of delirium.* How many instances of severe or fatal peritonitis puerperalis has Dr. P. seen unaccompanied by delirium? We would say, indeed, that inflammation of the serous membranes of the abdomen is far more productive of sensorial disturbance than hepatitis.

Dr. Philip next rehearses, from his work on Indigestion, his peculiar opinions respecting the *measure* which we are supposed to possess for habitual derangement of the biliary secretion, in "the fulness of the right side of the

* The reader is requested to peruse that section of Mr. Annesley's great work which treats of acute inflammation of the liver, or our analysis of it, in the 18th No. of this Journal for October, 1828, page 289, *et seq*. Let him there search for this pathognomonic symptom—delirium—and he will search in vain. We suspect that this is one of the many instances where Dr. Philip has mistaken reading or hearsay for those facts and personal observations on which his work is said to be solely based.

*body*"—" the consequence of a distended state of *that bowel into which the stomach first pours its contents.*" Here is a periphrasis with a vengeance, in order to avoid so learned a term as *duodenum*, which might not be intelligible to those who had made themselves masters of " the laws of the vital functions." Greatly do we wonder that Dr. Philip's hand did not tremble and his heart palpitate, when he felt himself thus lowering the language and the science of physic to the comprehension of general readers.

Dr. Philip assumes that this distention of the duodenum, and consequent fulness of the " right side of the body," is owing to bile less active in its properties than it should be. For our own parts, and we have paid some attention to the subject, we never could discover any other fulness in the right side of the abdomen, than what might be very easily accounted for by the ascending arch of the colon, and that part of the transverse arch which is near or in contact with the liver. These portions of the bowel are often the seats of accumulations—and much more likely to be so, and to produce inequality of the two sides than any accumulation in the duodenum. As for the state of the bile, we have reason to believe that it is as often acrimonious as it is inert—and that much more misery is produced by the draining of such bile into the upper portion of the intestinal tube, than by any imaginary distention of the duodenum, from insipidity of the hepatic secretion.* But the worthy Doctor having once got a theory into his head can never get it out again, notwithstanding all his professions about being solely a man of facts.

" All affections of the liver produce depression of spirits; hence the name melancholy. In its organic affection this symptom is generally more uniform; its secretion is also more uniformly deranged, although, as I have just had occasion to observe, it is not very uncommon to find the colour of the bile natural, even when the liver is both enlarged and indurated;—one among many proofs that, by the colour alone, we judge very imperfectly of the state of this fluid.

From the enlarged and, consequently, heavy liver pressing on other organs, the patient often experiences considerable difficulty in lying on the left side, although this symptom is not so uniform as, from reasoning, we might expect, and is probably influenced by the degree in which its ligaments are relaxed. When he lies on the right side, the enlarged liver resting only on the ribs and muscles, generally gives less uneasiness. As the disease advances he usually finds lying on the back the only easy posture; and in the most advanced cases, the easiest position is on the back, inclining a little to the right side with the shoulders considerably raised.

The symptoms, in the progress of the disease, vary according to the liability to disease in the particular constitution in those organs with which the liver chiefly sympathises, particularly the brain and lungs. In some cases the patient becomes more or less lethargic, the mind at times wandering; and long-continued irritation of the liver occasionally gives rise to some of those states which dispose to the different forms of apoplexy which we have been considering. Either by debilitating the vessels of the brain it gives a tendency to their morbid distension, or by its effect on the brain itself weakens its powers, and disposes to their failure from slight causes.

---

* Torpor of the lower bowels is far from being invariably dependent on torpor of the liver. A very considerable proportion of people, who labour under constipation of the bowels, have a perfectly sound state of the liver, and a healthy secretion of bile.

The headaches of bilious subjects every one has witnessed. They are, for the most part, felt chiefly in the forehead. They are of various descriptions; dull and heavy, or acute and lancinating, and not unfrequently occupy other parts of the head, the crown, the sides, or the back part.

In the same patient they generally return to the same part, and often excite the idea that there must be some fixed disease of the brain in the situation they occupy; but the pain is not constant, and although any uniformity in such symptoms is never favourable, this has very rarely, in my practice, proved to be the case. Some peculiar nervous sympathy, little inclined to produce organic disease, serves in bilious subjects to determine the particular seat of the head-ache.

One of the most common and fatal effects on the head of disordered liver, is what is called *internal water of the head*, which is apt to appear under thirteen years of age, and is most frequent in infants. In this disease, as in many of the other diseases of children, we see the illustration of a principle which has an extensive influence on the phenomena of disease.". 197.

This "internal water of the head" is considered by Dr. Philip as "almost always the effect of disordered liver," and owing, he thinks, to languid inflammation of the brain. The only successful plan of treatment, he avers, must be based on the hepatic origin of the disease.

In the remainder of the volume, occupied with the means of preventing or remedying those states or conditions which precede organic disease, we see little, if anything, that calls for remark—certainly nothing that we can quote with advantage to our readers. To the general public (if they peruse it at all) the greater part of this volume may appear new; but it is quite evident that the author never meant it for professional perusal. He is too conscientious, indeed, to expect that his brethren, who are not overladen with riches, could afford to purchase his works twice over, even when changed in title. We regret exceedingly that we have been compelled to speak in somewhat disrespectful terms of this publication; but we appeal to every individual who may take the trouble to examine the book, whether or not we have characterized it justly. It is exceedingly unworthy of a pen from which so much valuable matter had previously flowed.

